# Comparative Prediction of Methane Production In Vitro Using Multiple Regression Model and Backpropagation Neural Network Based on Cornell Net Carbohydrate and Protein System

**DOI:** 10.3390/vetsci12111099

**Published:** 2025-11-18

**Authors:** Guanghui Yu, Zenghui Li, Ruilan Dong

**Affiliations:** College of Animal Science and Technology, Qingdao Agricultural University, No. 700 Changcheng Road, Chengyang District, Qingdao 266109, China; guanghuiyu@qau.edu.cn (G.Y.); lizenghui@stu.qau.edu.cn (Z.L.)

**Keywords:** artificial neural network, in vitro fermentation, methane production, prediction models

## Abstract

Methane (CH_4_) produced by methanogenic archaea during the rumen fermentation of feed carbohydrates leads to global warming and total energy loss. This study aims to compare the accuracy of multiple linear regression (MLR) models and backpropagation neural network (BPNN) in predicting ruminal CH_4_ production of the carbohydrate (Carbs) components of the Cornell Net Carbohydrate and Protein System (CNCPS) in mixed rations of beef cattle with different concentrate-to-forage (C/F) ratios. Two datasets were established using the in vitro fermentation method of Menke and Steingass. One of the datasets contained 60 mixed rations with C/F ratios of 30:70, 40:60, 50:50, 60:40, 70:30, 80:20, and 90:10, respectively, which were used to develop CH_4_ prediction models. Another dataset included 10 mixed rations with the same C/F ratios, which were used to validate and compare the accuracy of the prediction models. Results indicated that there was a significant multiple regression relationship between CH_4_ production and the Carbs-components (CA (sugars), CB_1_ (starch and pectin), CB_2_ (available cell wall), CC (unavailable cell wall)) of CNCPS (r^2^ = 0.91, *p* < 0.0001). An optimal BPNN model with 2 hidden-layer neuron nodes was established with the same variables (r^2^ = 0.93, *p* < 0.0001). The findings demonstrated that both MLR and BPNN models (*p* < 0.0001) were suitable for predicting CH_4_ production using the Carbs components (CA, CB_1_, CB_2_, CC) of CNCPS. However, compared with the MLR model, the BPNN model has a greater coefficient of determination (r^2^) value and concordance correlation coefficient (CCC), and a lower root mean square prediction error (RMSPE), demonstrating better prediction performance.

## Simple Summary

Methane (CH_4_) produced from rumen fermentation in cattle is a major agricultural source of greenhouse gas (GHG) emissions that contributes to global warming. Therefore, accurate prediction of CH_4_ production from this process is critical for developing national CH_4_ emission inventories and evaluating dietary strategies to mitigate GHG emissions. Based on the in vitro fermentation of CH_4_ production, this study established a large-scale experimental database, which included training set data and test set data, respectively. Prediction models for CH_4_ production in vitro were developed by using multiple linear regression (MLR) analysis and backpropagation neural network (BPNN) technique, with the carbohydrate (Carbs) components of the Cornell Net Carbohydrate and Protein System (CNCPS) in the mixed rations as the input variable. The root mean square prediction error (RMSPE), coefficient of determination (r^2^), and concordance correlation coefficient (CCC) of the MLR and the BPNN prediction models were evaluated and compared using the test set data. In conclusion, compared with the MLR model (RMSPE% = 3.42%, r^2^ = 0.91, CCC = 0.92), the BPNN model (RMSPE% = 2.29%, r^2^ = 0.93, CCC = 0.96) is more accurate in predicting the CH_4_ production during rumen fermentation.

## 1. Introduction

Methane (CH_4_) is an important greenhouse gas (GHG), with a 100-year warming potential 28 times that of carbon dioxide (CO_2_), contributing significantly to global climate change [1]. A total of 159 countries, along with the European Commission, have committed to the Global Methane Pledge, which aims to reduce anthropogenic CH_4_ emissions by at least 30% by the year 2030, relative to levels recorded in 2020 [2]. This initiative seeks to achieve the goal of limiting the increase in global temperatures to no more than 1.5 °C [3]. Agriculture contributes 40% of CH_4_ emissions resulting from human activities, making it the primary source of this potent and environmentally detrimental GHG. Approximately 80% of agricultural CH_4_ emissions are generated from enteric fermentation in ruminants [4]. It is worth noting that beef and dairy production systems contribute over 70% of livestock GHG emissions [5].

The importance of mitigating CH_4_ emission has been increasingly recognized, highlighting the urgent need for precise quantitative techniques to formulate effective strategies for mitigating climate change [6]. However, due to the limitations of measurement techniques and high costs, it is difficult to assess enteric CH_4_ emissions in a commercial setting [7]. To enhance the accuracy of predicting enteric CH_4_ emissions in cattle, numerous prediction models have been reported, including univariate linear regression models [8,9], multiple linear regression (MLR) models [8,10,11], nonlinear models [12], and dynamic mechanistic models [13,14]. These models investigate the factors influencing CH_4_ production by examining the underlying mechanisms of CH_4_ formation in rumen fermentation. They incorporate key variables such as dry matter intake (DMI) [8,9], gross energy intake (GEI) [9], and metabolizable energy intake (MEI) [8], neutral detergent fiber intake (NDFI) [8,11], acid detergent fiber intake (ADFI) [8,11], ether extract (EE) [10], and forage proportion [8], which can be utilized as predictors for estimating CH_4_ output. However, application of the empirical models [7] beyond the range of the data used for model development can result in errors in CH_4_ prediction [15]. The predictive performance of CH_4_ models improves with massive datasets for model development. Many studies have shown that CH_4_ production during rumen fermentation is related to the dietary carbohydrates consumed by cattle [16]. In the Cornell Net Carbohydrate and Protein system (CNCPS), the carbohydrate (Carbs) components are divided into four components of CA (sugars), CB_1_ (starch and pectin), CB_2_ (available cell wall), and CC (unavailable cell wall), based on the rate of degradation in the rumen [17]. Dong et al. [18] indicated that the Carbs-components of the CNCPS in mixed rations were closely correlated with CH_4_ production in rumen fermentation; however, the mixed rations in that study are limited to low concentrate-to-forage (C/F) ratios. The C:F ratios in feeds affects CH_4_ production in ruminants by shifting rumen fermentation patterns and proportions of volatile fatty acids (VFAs) [19].

The artificial neural network (ANN) offers a powerful alternative for predicting CH_4_ emissions in ruminants. ANNs capture the complexity of the rumen microbial fermentation system through its capacity for nonlinear mapping and self-learning [20]. Unlike traditional statistical models, ANNs effectively characterize nonlinear relationships between input variables and CH_4_ emissions without requiring a predetermined functional form [21]. Furthermore, ANNs demonstrate significant advantages when managing high-dimensional datasets [22]. Shadpour et al. [23] predicted CH_4_ emissions from dairy cattle through the application of multilayer perceptron neural networks. Their findings highlight the ability of ANN to identify intricate patterns in milk composition that are linked to CH_4_ emissions. Sahraei et al. [24] evaluated Long Short-Term Memory networks, which had an R^2^ of 0.88 and a mean bias error (MBE) of 13.55 ppm CH_4_, demonstrating promising potential for predicting CH_4_ emissions in dairy cows. It can be presumed that using ANN algorithms to develop models can optimize the CH_4_ prediction in ruminants from diet compositions.

The objective of the present study was to investigate the relationship between CH_4_ production and the Carbs-components of the CNCPS in mixed rations of beef cattle with a wide range of C/F ratios. Additionally, an artificial neural network model based on backpropagation was developed using the CNCPS Carbs-components as input variables to determine whether the accuracy of the CH_4_ prediction models could be further improved.

## 2. Materials and Methods

This study comprises the following components: (1) Dataset construction: Two datasets were constructed for model development. (2) Variable selection: The CNCPS Carbs -components of various mixed rations were selected as independent variables of the model, with in vitro CH_4_ production as the dependent variable. (3) Model development: Two modeling approaches of a multiple linear regression (MLR) model and a backpropagation neural network (BPNN) were developed. (4) Model evaluation: The predictive performance of the developed models was validated and compared.

### 2.1. Construction of Two Datasets

Two independent datasets were constructed for modeling and validation. The modeling dataset used for simulation training included 60 mixed rations with C/F ratios of 30:70, 40:60, 50:50, 60:40, 70:30, 80:20, and 90:10, respectively (Table 1). The validation dataset was used to evaluate model performance, comprising 10 mixed rations with the same C/F ratios (Table 2). These dietary formulations were designed based on the ingredient compositions and C/F ratios commonly used in typical feeding practices for beef cattle in China [18].

### 2.2. In Vitro Fermentation

The in vitro CH_4_ production of mixed rations was determined by the in vitro fermentation method of Menke and Steingass [25]. Four Simmental bulls fitted with permanent rumen fistulas (Anscitech Co., Ltd., Wuhan, China) were used as donors of rumen fluid. The daily ration for the cattle consisted of 24.0 kg WCS and 4.0 kg concentrate mixture (as-fed basis). The concentrate was composed of 60% corn, 18% soybean meal, 13% wheat bran, 2% calcium hydrogen phosphate, 1% sodium chloride, 1% sodium bicarbonate, and 5% trace element mixture. The management of the cattle was conducted according to the Laboratory Animal Ethics Committee of the College of Animal Science and Technology at Qingdao Agricultural University (Approval No. DKY20240420). Two hours before the morning feeding, 100 mL of rumen fluid was collected from each cattle via rumen fistulas. The rumen fluid of the four bulls was thoroughly mixed and quickly filtered through four layers of gauze into a warmed bottle maintained at 39 °C. The filtered rumen fluid was mixed with artificial buffered saliva [25] in a ratio of 1:2 to prepare the rumen fluid–buffer mixture. The 100 mL calibrated glass syringes were used as the fermentation vessels. Each syringe was filled with 0.2000 g of the mixed ration, and the syringes were prewarmed to 39 °C before use. Four syringes were designated as replicates for each mixed ration within each experimental run. A total of three independent fermentation runs were conducted. Each fermentation syringe was loaded with 30 mL of 1:2 rumen fluid–buffer mixture. The syringes were flushed with CO_2_ to remove air, and the heads of the syringes were sealed. The syringes were then incubated on a rotary shaker platform in a water bath maintained at 39 °C for 48 h. The cumulative gas production of the mixed ration was recorded, and the pH of the fermentation broth was determined using a digital pH meter after 48 h of fermentation. After 48 h of incubation, a 20 mL gas sample was collected through a three-way plug valve between the sampling syringe and the incubation syringe to determine the gas concentration. The CH_4_ concentrations in the gas samples were analyzed by gas chromatography (TP-2060T, Beijing Beifen Tianpu Instrument Technology Co., Ltd., Beijing, China). The chromatographic conditions, including the TCD detector, carrier gas, and standard gas composition, were consistent with those previously described by Dong and Zhao [18]. The analysis was conducted using the following conditions: a TCD detector and a TDX-01 column (dimensions 1 m × 2 mm × 3 mm) were used, with the column maintained at 70 °C and the detector at 100 °C. Argon was used as the carrier gas at a flow rate of 30 mL/min. The standard gas included 26.796% CH_4_, 65.300% CO_2_, 0.605% O_2_, 7.100% N_2_, and 0.199% H_2_ (*v*/*v*).

### 2.3. Chemical Analysis of the Mixed Ration

The chemical composition of dry matter (DM), ether extract (EE), CP, and ash contents of the feed samples were analyzed using method 930.15, 984.13, 920.39 and 942.05 of AOAC [26]. Starch content was determined as described by Dong and Zhao [18] with an enzyme kit containing thermostable α-amylase and amyloglucosidase (Megazyme International Ireland Ltd., Wicklow, Ireland). The neutral detergent fiber (NDF) content (method 2002.04) was determined using the ANKOM fiber system (ANKOM Technology, Macedon, NY, USA). Neutral detergent insoluble crude protein (NDICP) was quantified as the crude protein content in the residues following NDF analysis. During the determination process, heat-stable α-amylase (ANKOM Technology) and sodium sulfite (ANKOM Technology) were used. The acid detergent lignin contents were determined using method 973.18 of AOAC [26].

### 2.4. Calculations

The CNCPS Carbs-components CA (sugars), CB_1_ (starch and pectin), CB_2_ (available cell wall), and CC (unavailable cell wall) of the mixed rations were determined using the method of Sniffen et al. [17], and presented in Table 3 and Table 4, respectively.

CA [%DM] = NSC [%DM] − Starch [%DM](1)

CB_1_ [%DM] = Starch [%DM] (2)

CB_2_ [%DM] = NDF [%DM] − NDICP [%DM] − CC [%DM] (3)

CC [%DM] = Lignin [%DM] × 2.4(4)

NSC [%DM] = 100 − CP [%DM] − Ash [%DM] − EE [%DM] − CB_2_ [%DM] − CC [%DM] (5)

where NSC refers to non-structural carbohydrate, and NDICP is neutral detergent insoluble crude protein.

CH_4_ concentration from the syringe was corrected for blanks and expressed as the amount of substrate dry matter (mL CH_4_/g DM). The CH_4_ production was calculated using the equation as follows:

(6) $$MP={MP}_{total}-{MP}_{blank}$$

where ${MP}_{total}$ refers to CH_4_ production of mixed rations after 48 h, and ${MP}_{blank}$ is CH_4_ production of the blank after 48 h of incubation.

### 2.5. Construction of MLR Prediction Models

The Carbs-components CA (sugars), CB_1_ (starch and pectin), CB_2_ (available cell wall), and CC (unavailable cell wall) of CNCPS of the dataset containing 60 mixed rations were used as independent variables for model input, and the in vitro CH_4_ production corresponding to 60 mixed rations was used as dependent variable for model output. The multiple regression relationship between the in vitro CH_4_ production (mL/g DM) and Carbs-components (%DM) of CNCPS was investigated using the following equation:

(7) $$y=a_{1}\left(CA\right)+a_{2}({CB}_{1})+a_{3}({CB}_{2})+a_{4}(CC)+b$$

where *y* refers to in vitro CH_4_ or CO_2_ production; bis the constant; and $a_{1}$, $a_{2}$, $a_{3}$ and $a_{4}$ are coefficients.

### 2.6. Construction of BPNN Prediction Models

A three-layer backpropagation neural network (BPNN) architecture including input layer neurons, hidden layer neurons, and output layer neurons was used in this study [27]. The input layer neurons are composed of four variables, CA, CB_1_, CB_2_ and CC. The hidden layer structure is a single monolayer with a different number of neurons. The output layer neuron contains an output variable for in vitro CH_4_ or CO_2_ production.

The BPNN model was developed on the MATLAB R2024a platform (The Math Works, Natick, MA, USA, 2024) using in vitro CH_4_ or CO_2_ production and CNCPS Carbs-components, which were derived from a training dataset containing 60 mixed rations. The training parameters included a learning rate of 0.1, training epochs of 1000, and a performance goal of 0.00001. The codes were runed in MATLAB R2024a as follows:

>> Independent variable_train = Independent variable_train’;
>> Dependent variable_train = Dependent variable_train’;
>> Independent variable_validation = Independent variable_validation’;
>> net = newff (Independent variable_train, Dependent variable_train, hidden layer number);
>> net.trainParam.epochs = 1000;
>> net.trainParam.lr = 0.1;
>> net.trainParam.goal = 0.00001;
>> net = train(net, Independent variable_train, Dependent variable_train);
>> Predicted Dependent variable_validation = sim(net, Independent variable_validaton);
>> Predicted Dependent variable_validation

The prediction performance of BPNN architectures with 1 to 20 neurons in the hidden layer was evaluated using the validation dataset.

### 2.7. Comparison of the Predictive Performance of MLR and BPNN

The validation dataset included in vitro CH_4_ or CO_2_ production and the CNCPS Carbs-components of 10 other mixed rations for evaluating the MLR models and BPNN models. The performance of the model was evaluated using the following four methods:

A *t*-test was used to compare the observed and the predicted values;

A linear regression relationship between the observed and the predicted values was analyzed using the following equation:

(8) y=bx+a

where *x* and *y* refer to the observed and predicted values, mL/g DM of mixed rations.

The root mean square prediction error (MSPE) [28] was calculated to quantify the deviation between the observed and the predicted values:

(9) $$MSPE=\frac{1}{n}\sum_{i=1}^{n}{\left({\hat{O}}_{i}-{\hat{P}}_{i}\right)}^{2}$$

(10) $$RMSPE\%=\sqrt{MSPE}/average\;observed\;value\times 100$$

where *i* = 1, 2, …, *n*; ${\hat{O}}_{i}$ refers to the observed value; ${\hat{P}}_{i}$, the predicted value; and n, the number of determinations. RMSPE is the ratio of the observed mean used to indicate the overall prediction error, %;

The concordance correlation coefficient (CCC) was calculated according to the formula reported by Lin [29].

### 2.8. Statistical Analysis

Statistical analysis was performed using SAS version 9.4 (SAS Institute Inc., Cary, NC, USA). Multiple linear regression (MLR) relationships between the dependent and independent variables were analyzed by using the PROC MIXED procedure. Selection of independent variables for the CH_4_ prediction equation was based on stepwise regression analysis, and only those with statistical significance (*p* ≤ 0.05) were retained. The assessment of multicollinearity among independent variables was conducted using the “vif” function, with the tolerance level set at a variance inflation factor <5.0. The *t*-test was used to compare the observed and predicted values, and the PROC MIXED procedure was used to analyze the linear relationship between them.

## 3. Results

### 3.1. The CH_4_ and CO_2_ Productions Through In Vitro Fermentation

The in vitro CH_4_ production for the 60 mixed rations with varying concentrate-to-forage ratios used for model development was presented in Table 5. After 48 h in vitro fermentation, the pH values of the fermentation residues were within the range of 6.10 to 6.80, indicating normal in vitro ruminal fermentation.

### 3.2. The Multiple Regression Relationship Between CH_4_ Emissions and CNCPS C-Components

The significant multiple regression equation between ruminal CH_4_ production in vitro and the Carbs-components CA (sugars), CB_1_ (starch and pectin), CB_2_ (available cell wall), and CC (unavailable cell wall) of the CNCPS was as follows:

CH_4_ (mL/g DM) = (0.22 ± 0.08) CA + (0.32 ± 0.05) CB_1_ − (0.13 ± 0.05) CB_2_ − (0.49 ± 0.09) CC + (34.05 ± 4.20) (R^2^ = 0.85, n = 60, *p* < 0.0001, MLR model) (11)

The significant multiple regression equation between ruminal CO_2_ production in vitro and the Carbs-components CA, CB_1_, CB_2_, and CC of the CNCPS was as follows:

CO_2_ (mL/g DM) = (2.34 ± 0.34) CA + (3.27 ± 0.24) CB_1_ + (0.44 ± 0.23) CB_2_ − (1.75 ± 0.41) CC + (36.35 ± 18.27) (R^2^ = 0.93, n = 60, *p* < 0.0001, MLR model) (12)

### 3.3. The BPNN Model

**Figure 1:**
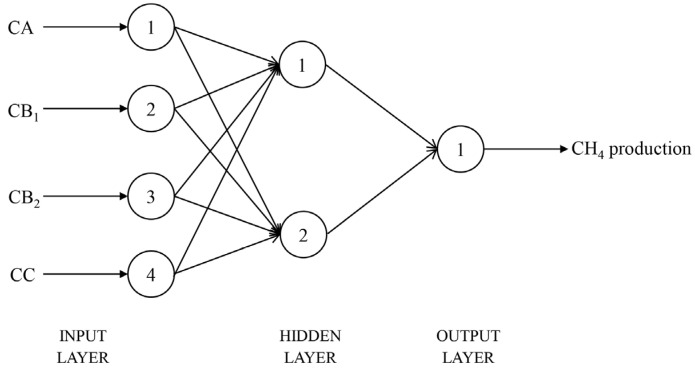
Structural diagram of the BPNN model for CH_4_ production prediction (CA = sugars, CB_1_ = starch and pectin, CB_2_ = available cell wall, CC = unavailable cell wall).

**Figure 2:**
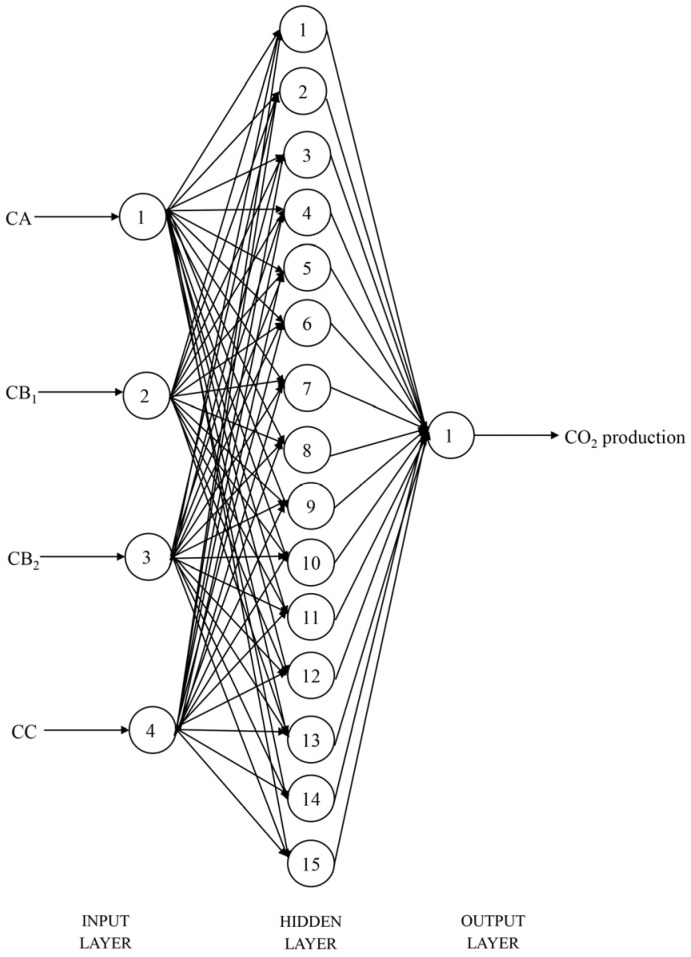
Structural diagram of the backpropagation neural network model for CO_2_ production prediction (CA = sugars, CB_1_ = starch and pectin, CB_2_ = available cell wall, CC = unavailable cell wall).

**Figure 3:**
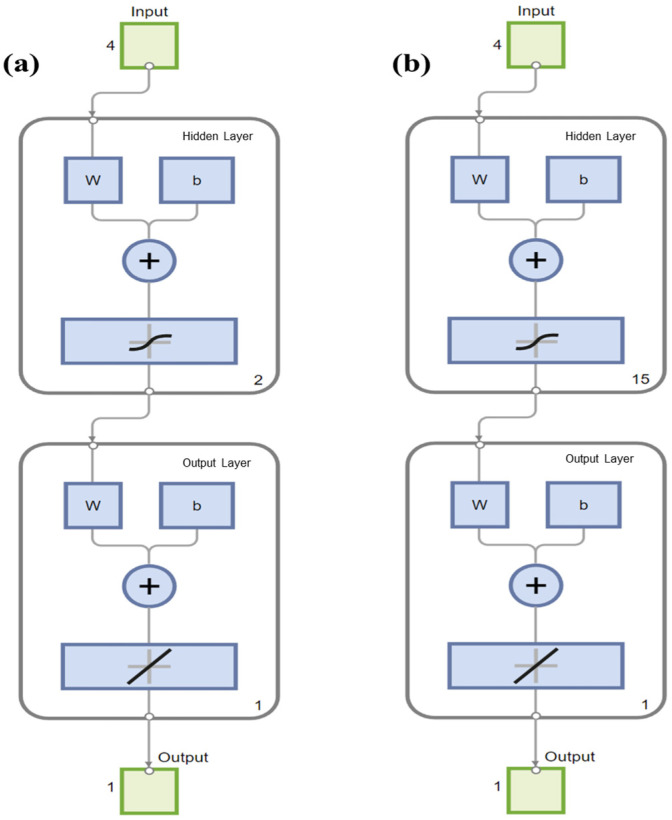
Backpropagation neural network models for (**a**) CH_4_ production and (**b**) CO_2_ production running on MATLAB.

**Figure 4:**
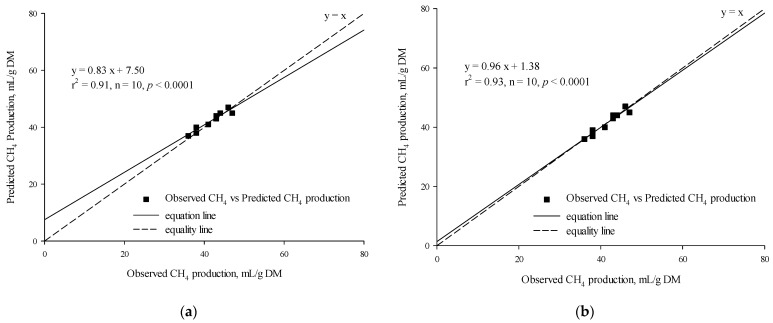
Linear relationship between the observed vs. the predicted (**a**) CH_4_ production from the multiple linear regression model and (**b**) CH_4_ production from the backpropagation neural network model.

After 1000 training epochs, the target error of 0.00001 was achieved. The RMSPE% of the BPNN model in predicting in vitro CH_4_ and CO_2_ production, with the number of hidden layer neuron nodes ranging from 1 to 20, is shown in Table 6. When the number of neuron nodes in the hidden layer was 2 and 15, respectively, the RMSPE% values for predicting the in vitro CH_4_ and CO_2_ production were the lowest. Therefore, the BPNN architectures suitable for predicting the in vitro CH_4_ and CO_2_ production were identified as 4-2-1 (Figure 1) and 4-15-1 (Figure 2), respectively. The BPNN models for predicting in vitro CH_4_ and CO_2_ production are presented in Figure 3.

### 3.4. Comparative Validation of the MLR and BPNN Models

The *t*-test results indicated that there was no statistically significant difference between the observed and predicted in vitro CH_4_ production for either the MLR (*p* = 0.14, Table 7) or the BPNN model (*p* = 0.76, Table 7). There was a significant linear regression relationship between the observed in vitro CH_4_ production and the predicted in vitro CH_4_ production based on the MLR (r^2^ = 0.91, *p* < 0.0001, n = 10; Figure 4a) and the BPNN model (r^2^ = 0.93, *p* < 0.0001, n = 10; Figure 4b). The RMSPE% and CCC values of the MLR and BPNN models were 3.42% vs. 2.29% and 0.92 vs. 0.96, respectively.

**Figure 5:**
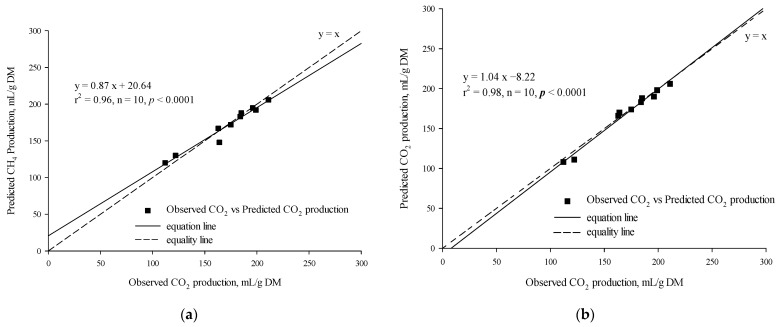
Linear relationship between the observed vs. the predicted (**a**) CO_2_ production from the multiple linear regression model and (**b**) CO_2_ production from the backpropagation neural network model.

The *t*-test results revealed no statistically significant difference between the observed and predicted in vitro CO_2_ production for either the MLR model (*p* = 0.68) or the BPNN model (*p* = 0.31). A strong linear regression relationship was found between the observed in vitro CO_2_ production and the predicted in vitro CO_2_ production using the MLR model (*r*^2^ = 0.96, *p* = 0.0046, n = 10; Figure 5a) and the BPNN model (r^2^ = 0.98, *p* = 0.0046, n = 10; Figure 5b). The RMSPE% values of the MLR and BPNN models were 4.13% and 2.95%, while their CCC values were 0.97 and 0.99, respectively.

## 4. Discussion

Since in vitro gas production is closely related to enteric CH_4_ yield in vitro (*r* = 0.88), the in vitro rumen fermentation technique can be used as a dynamic estimation tool to predict enteric CH_4_ emissions in vitro [30]. In addition, measuring enteric CH_4_ emissions through the in vitro rumen fermentation method can facilitate the development of a large dataset essential for accurate CH_4_ prediction models. A pH range of 6.0 to 6.8 is optimal for maintaining the stability of the rumen’s internal conditions [31]. In this study, the pH value of the incubation residues fell within the normal range. Furthermore, rumen microorganisms were found to remain active after the in vitro fermentation, indicating that the in vitro incubation system was effectively functioning.

The carbohydrate components of ruminant feeds in the CNCPS reflect the fermentation degradability in rumen [17]. In the current study, there was a significant multiple regression relationship between the CNCPS-Carbs components in mixed rations with varying C/F ratios and the in vitro CH_4_ production, with an R^2^ of 0.85. This regression determination coefficient is consistent with the multiple regression equation reported by Wang et al. [11]. The results demonstrated that the CA, CB_1_, CB_2_, and CC four components in CNCPS are suitable variables for predicting CH_4_ production of mixed rations with different C/F ratios. In addition, the coefficients of CA, CB_1_, and CB_2_ in the MLR prediction model were 0.22, 0.32, and 0.13 mL/g DM, respectively, demonstrating that CB_2_ had a lower CH_4_ production compared with CA and CB_1_. This finding is consistent with the previous work of Dong et al. (2013) [18]. Selecting the appropriate predictor variables could enhance the model’s prediction effect on CH_4_ production. A linear mixed model established by Oikawa et al. [32] that included DMI, BW, and milk fat as predictors had an R^2^ of 0.66 and a CCC of 0.66. One possible reason for the low accuracy of that study might be that the dataset is limited to typical feed ingredients from Japan. Vargas et al. [33] evaluated the predictive performance of 72 models for predicting CH_4_ emissions in beef cattle, and found that the RMSPE value of the Ellis et al. [8] model (including the prediction variables of DMI and EE) was as low as 17.79%, and the CCC value was 0.21. The MLR model established in this study could be used in cattle production to identify low CH_4_ emitting feed formulas [34]. By manipulating the proportions of CNCPS components, producers can pre-emptively design rations that not only meet the nutritional requirements of animals but also reduce CH_4_ emissions.

In recent years, researchers have applied deep learning methods to develop prediction models to enhance the accuracy of CH_4_ prediction [35]. The enteric CH_4_ produced from rumen fermentation of ruminants is a complex and dynamic reaction process [36]. The ANN algorithms provide advanced tools for dynamically simulating complex relational models [37]. Most studies apply ANNs to predict parameters in various fields to help further improve the accuracy of the model development. Jassim et al. [38] developed a multi-layer perceptron artificial neural network to predict municipal solid waste generation, with an R^2^ value of 0.94. Odufuwa et al. [39] evaluated emissions of pollutants such as CO_2_ and nitrogen oxides (NO_x_). The ANN model demonstrated outstanding accuracy, with a regression (R) value exceeding 0.9 and a mean square error (MSE) as low as 0.0046 under a multiple training-validation-test configuration. Louime et al. [40] designed a convolutional Neural Network (CNN) model to accurately predict CH_4_ emissions from seaweed. In this study, BPNN was used to develop the CH_4_ production model and demonstrated superior predictive performance (*r*^2^ = 0.94). Sahraei et al. [24] developed a deep learning model that used a moderate and public dataset to predict CH_4_ productions from enteric fermentation in dairy cows, substantially improving the model performance (R^2^ = 0.74). There may be a nonlinear relationship between CH_4_ emissions and production traits, as Shadpour et al. [23] found that compared with linear ANNs and partial least squares (PLS) regression, nonlinear artificial neural networks (ANNs) had better predictive performance. In this study, the BPNN models are developed based on a nonlinear function of the Levenberg–Marquardt algorithm. Each neuron in the input layer is connected to the neurons of the hidden layer and then linked to the output layer through connection weighs [41]. Therefore, the number of neuron nodes in the hidden layer may affect the learning efficiency of the neural network and subsequently influence the prediction performance of the model. In this study, a different number of neurons in the hidden layer ranging from 1 to 20 were trained in the BPNN models, and the RMSPE showed some fluctuations with different neuron nodes. The results indicated that the BPNN with two hidden layer nodes can reduce CH_4_ emissions by 2.12%, which has great potential in the real-time application of dynamic rumen microbial fermentation. This indicates that ≥3 neurons in the hidden layer will lead to overfitting of the neural network, while only 1 neuron in the hidden layer will result in insufficient training of the neural network. It should be noted that the limitation of this study lies in the relatively small sample size of the validation dataset (n = 10). In future research, its predictive performance still needs to be further tested in larger databases.

The accuracy of BPNN and MLR models in predicting enteric CH_4_ and CO_2_ productions in vitro was compared using the validation dataset of this study. It revealed that the *r*^2^ and CCC values of the BPNN model were greater than those of the MLR model, while the RMSPE value was lower than that of the MLR model. The results show that when the four CNCPS Carbs-components were available, the BPNN approach outperforms the MLR model, demonstrating the potential of artificial intelligence in predicting enteric CH_4_ emissions in cattle. This finding is consistent with the predicted ruminal ammonia-N [27], indicating that the ANN algorithm has superior overall performance in predicting rumen fermentation parameters. Similarly, Stamenković et al. [42] demonstrated that the predictive performance of the ANN model was much better than that of the conventional MLR model when predicting CH_4_ emission in 20 European countries. Previous studies have reported that the ANN model performed better than the random forest (RF) model [43], and the performance of BPNN is better than that of the nonlinear regression models [44]. It is reported that the machine learning method (R^2^ ≥ 0.61) outperforms the linear method (R^2^ ≤ 0.56) in predicting CH_4_ production from anaerobic digestion of lignocellulosic biomass [45]. Due to its powerful nonlinear capabilities and inherent self-learning characteristics of complex systems, ANN models have become important tools for predicting enteric CH_4_ emissions of ruminants [46]. Compared with traditional empirical regression models, neural networks can effectively capture the complex relationships between input variables and CH_4_ emissions without the need to predefine the functional forms. It lays the foundation for incorporating ANN technique into future sustainable environmental strategies and promotes the development of carbon reduction technologies. In addition to its predictive ability, the BPNN model for predicting CH_4_ productions established in this study has significant potential in biological applications. As a computer simulator, it can accelerate pre-screen the CH_4_ mitigation potential of dietary interventions before conducting costly in vivo trials. In addition, this model offers a new approach for precise feeding of ruminants, allowing for the formulation of dietary strategies based on predicted CH_4_ emission phenotypes. In the future, additional research is necessary to integrate the microbial community, genotypes, and production data to develop highly robust models for accurately estimating enteric CH_4_ emissions from cattle [47].

## 5. Conclusions

There is a significant multiple linear regression relationship between the enteric CH_4_ production in vitro and the CNCPS Carbs-components (CA, CB_1_, CB_2_, and CC) within varying C/F ratios of mixed rations. The BPNN model can accurately simulate the intrinsic relationship between the carbohydrate composition of feed and CH_4_ production during ruminal anaerobic fermentation. The *r*^2^ and CCC values are relatively large, while the RMSPE value is relatively low. This method provides an artificial predictive tool for cattle farming, enabling pre-emptive diet design that not only meets the nutritional requirements of animals but also reduces CH_4_ emissions.

## Figures and Tables

**Table 1:** Typical mixed rations for beef cattle used to establish models with concentrate-to-forage ratios (C/F) ranging from low to high (%, dry matter basis).

Ration No.	CG	SM	WB	CM	RM	DDGS	CS	AH	CW	RS	WS	WCS	C/F
1	20	12	6.0	—	—	2.0	—	—	—	—	—	60	40:60
2	25	15	7.5	—	—	2.5	—	—	—	—	—	50	50:50
3	30	18	9.0	—	—	3.0	—	—	—	—	—	40	60:40
4	35	21	10.5	—	—	3.5	—	—	—	—	—	30	70:30
5	40	24	12.0	—	—	4.0	—	—	—	—	—	20	80:20
6	45	27	13.5	—	—	4.5	—	—	—	—	—	10	90:10
7	15	9	4.5	—	—	1.5	70	—	—	—	—	—	30:70
8	25	15	7.5	—	—	2.5	50	—	—	—	—	—	50:50
9	30	18	9.0	—	—	3.0	40	—	—	—	—	—	60:40
10	35	21	10.5	—	—	3.5	30	—	—	—	—	—	70:30
11	40	24	12.0	—	—	4.0	20	—	—	—	—	—	80:20
12	45	27	13.5	—	—	4.5	10	—	—	—	—	—	90:10
13	16.5	5.7	5.4	2.4	—	—	—	28	—	—	—	42	30:70
14	22	7.6	7.2	3.2	—	—	—	24	—	—	—	36	40:60
15	33	11.4	10.8	4.8	—	—	—	16	—	—	—	24	60:40
16	38.5	13.3	12.6	5.6	—	—	—	12	—	—	—	18	70:30
17	44	15.2	14.4	6.4	—	—	—	8.0	—	—	—	12	80:20
18	49.5	17.1	16.2	7.2	—	—	—	4.0	—	—	—	6	90:10
19	16.5	5.7	5.4	2.4	—	—	—	—	70	—	—	—	30:70
20	22	7.6	7.2	3.2	—	—	—	—	60	—	—	—	40:60
21	27.5	9.5	9.0	4.0	—	—	—	—	50	—	—	—	50:50
22	38.5	13.3	12.6	5.6	—	—	—	—	30	—	—	—	70:30
23	44	15.2	14.4	6.4	—	—	—	—	20	—	—	—	80:20
24	49.5	17.1	16.2	7.2	—	—	—	—	10	—	—	—	90:10
25	16.8	4.5	4.8	3.0	0.9	—	—	—	—	70	—	—	30:70
26	22.4	6.0	6.4	4.0	1.2	—	—	—	—	60	—	—	40:60
27	28	7.5	8.0	5.0	1.5	—	—	—	—	50	—	—	50:50
28	33.6	9.0	9.6	6.0	1.8	—	—	—	—	40	—	—	60:40
29	44.8	12	12.8	8.0	2.4	—	—	—	—	20	—	—	80:20
30	50.4	13.5	14.4	9.0	2.7	—	—	—	—	10	—	—	90:10
31	16.8	4.5	4.8	3.0	0.9	—	—	—	—	—	70	—	30:70
32	22.4	6.0	6.4	4.0	1.2	—	—	—	—	—	60	—	40:60
33	28	7.5	8.0	5.0	1.5	—	—	—	—	—	50	—	50:50
34	33.6	9.0	9.6	6.0	1.8	—	—	—	—	—	40	—	60:40
35	39.2	10.5	11.2	7.0	2.1	—	—	—	—	—	30	—	70:30
36	50.4	13.5	14.4	9.0	2.7	—	—	—	—	—	10	—	90:10
37	15	3.9	4.5	1.8	1.8	3.0	—	—	—	—	28	42	30:70
38	20	5.2	6.0	2.4	2.4	4.0	—	—	—	—	24	36	40:60
39	25	6.5	7.5	3.0	3.0	5.0	—	—	—	—	20	30	50:50
40	30	7.8	9.0	3.6	3.6	6.0	—	—	—	—	16	24	60:40
41	35	9.1	10.5	4.2	4.2	7.0	—	—	—	—	12	18	70:30
42	40	10.4	12.0	4.8	4.8	8.0	—	—	—	—	8	12	80:20
43	15	3.9	4.5	1.8	1.8	3.0	—	—	—	28	—	42	30:70
44	20	5.2	6.0	2.4	2.4	4.0	—	—	—	24	—	36	40:60
45	25	6.5	7.5	3.0	3.0	5.0	—	—	—	20	—	30	50:50
46	30	7.8	9.0	3.6	3.6	6.0	—	—	—	16	—	24	60:40
47	40	10.4	12	4.8	4.8	8.0	—	—	—	8	—	12	80:20
48	45	11.7	13.5	5.4	5.4	9.0	—	—	—	4	—	6	90:10
49	17.1	6.0	5.1	—	1.8	—	—	70	—	—	—	—	30:70
50	22.8	8.0	6.8	—	2.4	—	—	60	—	—	—	—	40:60
51	28.5	10	8.5	—	3.0	—	—	50	—	—	—	—	50:50
52	34.2	12	10.2	—	3.6	—	—	40	—	—	—	—	60:40
53	39.9	14	11.9	—	4.2	—	—	30	—	—	—	—	70:30
54	51.3	18	15.3	—	5.4	—	—	10	—	—	—	—	90:10
55	17.1	6.0	5.1	—	1.8	—	28	—	—	—	—	42	30:70
56	22.8	8.0	6.8	—	2.4	—	24	—	—	—	—	36	40:60
57	28.5	10	8.5	—	3.0	—	20	—	—	—	—	30	50:50
58	34.2	12	10.2	—	3.6	—	16	—	—	—	—	24	60:40
59	39.9	14	11.9	—	4.2	—	12	—	—	—	—	18	70:30
60	45.6	16	13.6	—	4.8	—	8	—	—	—	—	12	80:20

Note: CG = corn grain, SM = soybean meal, WB = wheat bran, CM = cottonseed meal, RM = rapeseed meal, DDGS = distiller’s dried corn grains with solubles, CS = corn stover, AH = alfalfa hay, CW = Chinese wildrye, RS = rice straw, WS = wheat straw, WCS = whole-crop corn silage, C/F = concentrate-to-forage ratios. — indicates unavailable data.

**Table 2:** Typical mixed rations for beef cattle used to validate models with concentrate-to-forage ratios (C/F) ranging from low to high (%, dry matter basis).

Ration No.	CG	SM	WB	CM	RM	DDGS	CS	AH	CW	RS	WS	WCS	C/F
1	15	9	4.5	—	—	1.5	—	—	—	—	—	70	30:70
2	20	12	6	—	—	2	60	—	—	—	—	—	40:60
3	27.5	9.5	9	4	—	—	—	20	—	—	—	30	50:50
4	33	11.4	10.8	4.8	—	—	—	—	40	—	—	—	60:40
5	39.2	10.5	11.2	7	2.1	—	—	—	—	30	—	—	70:30
6	44.8	12	12.8	8	2.4	—	—	—	—	—	20	—	80:20
7	45	11.7	13.5	5.4	5.4	9	—	—	—	—	4	6	90:10
8	35	9.1	10.5	4.2	4.2	7	—	—	—	12	—	18	70:30
9	45.6	16	13.6	—	4.8	—	—	20	—	—	—	—	80:20
10	51.3	18	15.3	—	5.4	—	4	—	—	—	—	6	90:10

Note: CG = corn grain, SM = soybean meal, WB = wheat bran, CM = cottonseed meal, RM = rapeseed meal, DDGS = distiller’s dried corn grains with solubles, CS = corn stover, AH = alfalfa hay, CW = Chinese wildrye, RS = rice straw, WS = wheat straw, WCS = whole-crop corn silage, C/F = concentrate-to-forage ratios. — indicates unavailable data.

**Table 3:** The CNCPS components (% DM) of typical rations for beef cattle used to develop models.

Ration No.	Carbohydrates	CNCPS Carbohydrate Components				NSC ^1^
		CA	CB_1_	CB_2_	CC	
1	80.03	40.13	20.55	14.69	4.66	60.67
2	78.47	37.15	23.52	13.72	4.08	60.67
3	76.91	34.18	26.49	12.74	3.50	60.68
4	75.36	31.21	29.47	11.76	2.92	60.68
5	73.80	28.24	32.44	10.78	2.34	60.68
6	72.24	25.26	35.41	9.80	1.77	60.68
7	76.53	22.16	14.54	32.13	7.70	36.71
8	74.86	22.20	21.36	25.47	5.84	43.55
9	74.02	22.22	24.76	22.14	4.91	46.98
10	73.19	22.24	28.17	18.81	3.98	50.40
11	72.35	22.25	31.57	15.48	3.05	53.83
12	71.52	22.27	34.98	12.15	2.12	57.25
13	81.41	37.96	17.08	15.02	11.35	55.04
14	80.28	35.81	20.38	14.15	9.94	56.19
15	78.01	31.53	26.97	12.40	7.12	58.50
16	76.88	29.38	30.27	11.52	5.71	59.65
17	75.75	27.24	33.57	10.65	4.30	60.80
18	74.62	25.09	36.86	9.78	2.89	61.96
19	83.12	21.05	16.72	29.44	15.91	37.77
20	81.75	21.32	20.07	26.51	13.84	41.39
21	80.37	21.60	23.42	23.57	11.78	45.01
22	77.62	22.14	30.11	17.71	7.66	52.25
23	76.24	22.41	33.46	14.77	5.60	55.87
24	74.86	22.68	36.81	11.84	3.54	59.49
25	80.73	13.47	14.44	37.98	14.83	27.91
26	79.68	14.72	18.16	33.85	12.94	32.89
27	78.64	15.97	21.89	29.71	11.06	37.86
28	77.59	17.22	25.61	25.58	9.18	42.83
29	75.50	19.72	33.06	17.31	5.42	52.78
30	74.46	20.97	36.78	13.17	3.54	57.75
31	81.89	14.32	15.15	45.67	6.75	29.46
32	80.68	15.45	18.77	40.44	6.02	34.21
33	79.47	16.57	22.39	35.21	5.29	38.97
34	78.26	17.70	26.01	29.97	4.57	43.72
35	77.05	18.83	29.64	24.74	3.84	48.47
36	74.62	21.09	36.88	14.27	2.38	57.97
37	81.75	31.02	16.52	28.17	6.04	47.55
38	80.35	29.50	19.73	25.65	5.46	49.23
39	78.95	27.98	22.93	23.14	4.89	50.92
40	77.54	26.46	26.14	20.62	4.32	52.60
41	76.14	24.94	29.35	18.11	3.74	54.29
42	74.74	23.42	32.55	15.59	3.17	55.98
43	81.29	30.69	16.24	25.09	9.27	46.93
44	79.95	29.21	19.49	23.02	8.23	48.70
45	78.61	27.74	22.73	20.94	7.20	50.48
46	77.28	26.27	25.98	18.86	6.16	52.25
47	74.60	23.33	32.47	14.71	4.09	55.80
48	73.27	21.86	35.72	12.64	3.06	57.57
49	72.81	32.09	5.88	13.50	21.33	37.97
50	69.53	31.80	6.18	12.61	18.94	37.98
51	66.25	31.52	6.48	11.71	16.55	37.99
52	62.97	31.23	6.78	10.81	14.15	38.01
53	59.70	30.94	7.08	9.92	11.76	38.02
54	53.14	30.36	7.68	8.12	6.98	38.04
55	73.32	37.06	7.24	21.78	7.24	44.30
56	69.97	36.06	7.35	19.70	6.86	43.41
57	66.61	35.06	7.45	17.62	6.48	42.52
58	63.26	34.06	7.56	15.54	6.10	41.62
59	59.91	33.07	7.66	13.46	5.72	40.73
60	56.56	32.07	7.77	11.38	5.34	39.84

^1^ NSC refers to non-structural carbohydrate (%DM). CNCPS = Cornell Net Carbohydrate and Protein System, CA = sugars, CB_1_ = starch and pectin, CB_2_ = available cell wall, CC = unavailable cell wall.

**Table 4:** The CNCPS components (%DM) of typical rations for beef cattle used to validate models.

Ration No.	Carbohydrates	CNCPS Carbohydrate Components				NSC ^1^
		CA	CB_1_	CB_2_	CC	
1	81.58	43.10	17.58	15.67	5.24	60.67
2	75.69	22.18	17.95	28.80	6.77	40.13
3	79.15	33.67	23.67	13.27	8.53	57.34
4	78.99	21.87	26.77	20.64	9.72	48.63
5	76.55	18.47	29.33	21.44	7.30	47.80
6	75.83	19.96	33.26	19.51	3.11	53.22
7	73.33	21.91	35.76	13.08	2.60	57.66
8	75.94	24.80	29.22	16.79	5.13	54.02
9	56.42	30.65	7.38	9.02	9.37	38.03
10	53.21	31.07	7.88	9.31	4.96	38.95

^1^ NSC refers to non-structural carbohydrate (%DM). CNCPS = Cornell Net Carbohydrate and Protein System, CA = sugars, CB_1_ = starch and pectin, CB_2_ = available cell wall, CC = unavailable cell wall.

**Table 5:** The CH_4_, CO_2_ productions (mL/g DM), and pH values of typical rations for beef cattle used to develop models.

Ration No.	CH_4_	CO_2_	pH Value
1	46 ± 0	196 ± 2	6.37 ± 0.01
2	46 ± 1	195 ± 2	6.72 ± 0.01
3	47 ± 0	193 ± 2	6.80 ± 0.03
4	47 ± 1	192 ± 1	6.84 ± 0.01
5	51 ± 1	207 ± 1	6.48 ± 0.06
6	48 ± 0	211 ± 1	6.92 ± 0.01
7	40 ± 1	153 ± 0	6.48 ± 0.02
8	40 ± 1	185 ± 2	6.12 ± 0.01
9	42 ± 1	195 ± 1	6.23 ± 0.01
10	43 ± 1	199 ± 2	6.30 ± 0.02
11	45 ± 1	202 ± 1	6.37 ± 0.01
12	47 ± 1	217 ± 2	6.41 ± 0.03
13	45 ± 1	170 ± 1	6.19 ± 0.03
14	42 ± 1	182 ± 2	6.28 ± 0.02
15	45 ± 1	189 ± 2	6.44 ± 0.01
16	46 ± 1	197 ± 1	6.51 ± 0.03
17	47 ± 1	206 ± 1	6.58 ± 0.02
18	53 ± 1	199 ± 1	6.43 ± 0.01
19	30 ± 0	115 ± 1	6.19 ± 0.03
20	33 ± 0	131 ± 1	6.28 ± 0.02
21	36 ± 0	147 ± 1	6.36 ± 0.03
22	40 ± 1	180 ± 2	6.51 ± 0.03
23	43 ± 1	196 ± 2	6.58 ± 0.02
24	50 ± 1	212 ± 1	6.45 ± 0.01
25	34 ± 1	113 ± 2	6.44 ± 0.05
26	39 ± 1	130 ± 2	6.46 ± 0.02
27	35 ± 1	143 ± 2	6.56 ± 0.03
28	38 ± 1	159 ± 2	6.58 ± 0.03
29	44 ± 1	191 ± 1	6.61 ± 0.03
30	47 ± 1	200 ± 1	6.57 ± 0.02
31	31 ± 1	113 ± 1	6.35 ± 0.03
32	35 ± 1	125 ± 1	6.23 ± 0.02
33	37 ± 1	135 ± 2	6.16 ± 0.01
34	39 ± 1	165 ± 2	6.20 ± 0.02
35	41 ± 1	180 ± 2	6.18 ± 0.01
36	46 ± 1	211 ± 2	6.20 ± 0.02
37	41 ± 0	154 ± 2	6.24 ± 0.01
38	43 ± 1	156 ± 2	6.21 ± 0.01
39	43 ± 0	184 ± 1	6.17 ± 0.01
40	43 ± 1	190 ± 1	6.22 ± 0.03
41	45 ± 1	197 ± 1	6.28 ± 0.03
42	43 ± 1	204 ± 2	6.27 ± 0.01
43	38 ± 1	163 ± 1	6.39 ± 0.04
44	39 ± 0	172 ± 2	6.40 ± 0.01
45	40 ± 1	180 ± 2	6.42 ± 0.02
46	42 ± 1	183 ± 1	6.42 ± 0.01
47	45 ± 0	191 ± 1	6.50 ± 0.01
48	54 ± 0	191 ± 1	6.47 ± 0.02
49	30 ± 1	100 ± 2	6.33 ± 0.01
50	32 ± 1	103 ± 2	6.36 ± 0.01
51	33 ± 1	105 ± 2	6.36 ± 0.01
52	34 ± 0	107 ± 1	6.38 ± 0.01
53	35 ± 1	110 ± 2	6.40 ± 0.01
54	44 ± 1	114 ± 1	6.40 ± 0.01
55	38 ± 1	154 ± 2	6.32 ± 0.01
56	38 ± 0	148 ± 2	6.24 ± 0.02
57	38 ± 1	143 ± 1	6.23 ± 0.02
58	38 ± 1	138 ± 1	6.17 ± 0.01
59	38 ± 1	132 ± 1	6.19 ± 0.03
60	38 ± 1	127 ± 1	6.21 ± 0.01

Data were expressed as mean ± standard error (SE).

**Table 6:** RMSPE% and *r*^2^ values between observed and predicted values across different hidden layer nodes.

Number of Nodes	RMSPE%		*r* ^2^	
	CH_4_ Production	CO_2_ Production	CH_4_ Production	CO_2_ Production
1	5.12	4.97	0.79	0.93
2	2.29	9.62	0.93	0.81
3	4.83	6.81	0.83	0.87
4	3.42	3.87	0.88	0.95
5	9.57	3.33	0.09	0.97
6	3.42	5.12	0.84	0.93
7	4.54	5.89	0.76	0.93
8	2.88	4.88	0.90	0.95
9	6.16	3.71	0.63	0.98
10	2.43	3.22	0.93	0.97
11	3.81	4.94	0.81	0.95
12	9.31	3.26	0.28	0.98
13	9.60	3.33	0.38	0.98
14	4.47	3.87	0.79	0.95
15	3.06	2.95	0.91	0.98
16	8.47	3.06	0.18	0.98
17	6.25	9.45	0.71	0.74
18	4.65	5.18	0.78	0.94
19	7.25	4.90	0.51	0.93
20	8.16	7.26	0.25	0.84

**Table 7:** The CH_4_, CO_2_ productions (mL/g DM), and pH values of typical rations for beef cattle used to validate models.

Mixed Rations		CH_4_ Production			CO_2_ Production		pH Value
	Observed Values	MLR Predicted	BPNN Predicted	Observed Values	MLR Predicted	BPNN Predicted	
1	47 ± 0	45	45	199 ± 1	192	198	6.36 ± 0.03
2	38 ± 1	38	37	164 ± 1	148	170	6.47 ± 0.01
3	43 ± 1	43	43	184 ± 1	183	183	6.36 ± 0.03
4	38 ± 1	40	39	163 ± 1	167	166	6.44 ± 0.01
5	41 ± 1	41	40	175 ± 2	172	174	6.60 ± 0.02
6	44 ± 1	45	44	196 ± 2	195	190	6.15 ± 0.01
7	46 ± 1	47	47	211 ± 2	206	206	6.26 ± 0.03
8	43 ± 1	44	44	185 ± 2	188	188	6.43 ± 0.01
9	36 ± 1	37	36	112 ± 2	120	108	6.36 ± 0.01
10	38 ± 1	40	38	122 ± 1	130	111	6.22 ± 0.01

Data were expressed as mean ± standard error (SE). MLR = multiple linear regression, BPNN = backpropagation neural network.

## Data Availability

The original contributions presented in this study are included in the article. Further inquiries can be directed to the corresponding author.

## References

[1] Pachauri R.K., Meyer L.A., IPCC (2014). Climate Change 2014: Synthesis Report. Contribution of Working Groups I, II and III to the Fifth Assessment Report of the Intergovernmental Panel on Climate Change.

[2] Predybaylo E., Lelieveld J., Pozzer A., Gromov S., Zimmermann P., Osipov S., Klingmüller K., Steil B., Stenchikov G., McCabe M. (2025). Surface temperature and ozone responses to the 2030 Global Methane Pledge. Clim. Change.

[3] Masson-Delmotte V., Zhai P., Pörtner H.-O., Roberts D., Skea J., Shukla P.R., Pirani A., Moufouma-Okia W., Péan C., Pidcock R., IPCC (2018). Summary for Policymakers. Global Warming of 1.5 °C.

[4] CCAC (2021). Global Methane Assessment: Benefits and Costs of Mitigating Methane Emissions.

[5] Gerber P.J., Steinfeld H., Henderson B., Mottet A., Opio C., Dijkman J., Falcucci A., Tempio G. (2013). Tackling Climate Change Through Livestock: A Global Assessment of Emissions and Mitigation Opportunities.

[6] Beauchemin K.A., Kebreab E., Cain M., VandeHaar M.J. (2025). The path to net-zero in dairy production: Are pronounced decreases in enteric methane achievable?. Annu. Rev. Anim. Biosci..

[7] Hristov A.N., Ott T., Tricarico J., Rotz A., Waghorn G., Adesogan A., Dijkstra J., Montes F., Oh J., Kebreab E. (2013). Special topics—Mitigation of methane and nitrous oxide emissions from animal operations: III. A review of animal management mitigation options. J. Anim. Sci..

[8] Ellis J., Kebreab E., Odongo N., McBride B., Okine E., France J. (2007). Prediction of methane production from dairy and beef cattle. J. Dairy Sci..

[9] Ribeiro R., Rodrigues J., Maurício R., Borges A., e Silva R.R., Berchielli T., Valadares Filho S., Machado F., Campos M., Ferreira A. (2020). Predicting enteric methane production from cattle in the tropics. Animal.

[10] Donadia A.B., Torres R.N.S., Silva H.M.d., Soares S.R., Hoshide A.K., Oliveira A.S.d. (2023). Factors affecting enteric emission methane and predictive models for dairy cows. Animals.

[11] Wang Y., Song W., Wang Q., Yang F., Yan Z. (2024). Predicting enteric methane emissions from dairy and beef cattle using nutrient composition and intake variables. Animals.

[12] Patra A.K. (2017). Prediction of enteric methane emission from cattle using linear and non-linear statistical models in tropical production systems. Mitig. Adapt. Strateg. Glob. Change.

[13] Kass M., Ramin M., Hanigan M., Huhtanen P. (2022). Comparison of Molly and Karoline models to predict methane production in growing and dairy cattle. J. Dairy Sci..

[14] Muñoz-Tamayo R., Ruiz B., Blavy P., Giger-Reverdin S., Sauvant D., Williams S., Moate P. (2022). Predicting the dynamics of enteric methane emissions based on intake kinetic patterns in dairy cows fed diets containing either wheat or corn. Animal.

[15] Escobar-Bahamondes P., Oba M., Beauchemin K. (2016). Universally applicable methane prediction equations for beef cattle fed high-or low-forage diets. Can. J. Anim. Sci..

[16] Xue B., Thompson J.P., Yan T., Stergiadis S., Smith L., Theodoridou K. (2025). Dose–response effects of dietary inclusion of agro-industrial by-products on in vitro ruminal fermentation and methane production. J. Sci. Food Agric..

[17] Sniffen C.J., O’connor J., Van Soest P.J., Fox D.G., Russell J. (1992). A net carbohydrate and protein system for evaluating cattle diets: II. Carbohydrate and protein availability. J. Anim. Sci..

[18] Dong R., Zhao G. (2013). Relationship between the methane production and the CNCPS carbohydrate fractions of rations with various concentrate/roughage ratios evaluated using in vitro incubation technique. Asian-Australas J. Anim. Sci..

[19] Fouts J.Q., Honan M.C., Roque B.M., Tricarico J.M., Kebreab E. (2022). Enteric methane mitigation interventions. Transl. Anim. Sci..

[20] Li R., Xu A., Zhao Y., Chang H., Li X., Lin G. (2022). Genetic algorithm (GA)—Artificial neural network (ANN) modeling for the emission rates of toxic volatile organic compounds (VOCs) emitted from landfill working surface. J. Environ. Manag..

[21] Liu X., Tao F., Du H., Yu W., Xu K. (2020). Learning nonlinear constitutive laws using neural network models based on indirectly measurable data. J. Appl. Mech..

[22] Castillo-Girones S., Munera S., Martínez-Sober M., Blasco J., Cubero S., Gómez-Sanchis J. (2025). Artificial neural networks in agriculture, the core of artificial intelligence: What, when, and why. Comput. Electron. Agric..

[23] Shadpour S., Chud T.C., Hailemariam D., Plastow G., Oliveira H.R., Stothard P., Lassen J., Miglior F., Baes C.F., Tulpan D. (2022). Predicting methane emission in Canadian Holstein dairy cattle using milk mid-infrared reflectance spectroscopy and other commonly available predictors via artificial neural networks. J. Dairy Sci..

[24] Sahraei A., Knob D., Lambertz C., Gattinger A., Breuer L. (2025). Modeling enteric methane emission from dairy cows using deep learning approach. Sci. Total Environ..

[25] Menke K.H., Steingass H. (1988). Estimation of the energetic feed value obtained from chemical analysis and in vitro gas production using rumen fluid. Anim. Res. Dev..

[26] Association of Official Analytical Chemists (AOAC) (2005). Official Methods of Analysis.

[27] Dong R., Sun G., Yu G. (2022). Estimating in vitro ruminal ammonia-N using multiple linear models and artificial neural networks based on the CNCPS nitrogenous fractions of cattle rations with low concentrate/roughage ratios. J. Anim. Physiol. Anim. Nutr..

[28] Bibby J., Toutenburg H. (1977). Prediction and Improved Estimation in Linear Models.

[29] Lin L.I. (1989). A concordance correlation coefficient to evaluate reproducibility. Biometrics.

[30] Aboagye I.A., Rosser C.L., Baron V.S., Beauchemin K.A. (2021). In vitro assessment of enteric methane emission potential of whole-plant barley, oat, triticale and wheat. Animals.

[31] Liang J., Fang W., Chang J., Zhang G., Ma W., Nabi M., Zubair M., Zhang R., Chen L., Huang J. (2022). Long-term rumen microorganism fermentation of corn stover in vitro for volatile fatty acid production. Bioresour. Technol..

[32] Oikawa K., Terada F., Kurihara M., Suzuki T., Nonaka I., Hosoda K., Kamiya Y., Roh S., Haga S. (2025). Methane emission prediction models for lactating cows based on feed intake, body weight, and milk yield and composition: Variable methane conversion factor-based approach. J. Dairy Sci..

[33] Vargas J., Swenson M., Schilling-Hazlett A., Reis I., Velasquez C., Martins E., Sitorski L., Campos L., Carvalho P., Stackhouse-Lawson K. (2025). Evaluation of models of enteric methane emissions in finishing steers. Animal.

[34] Williams S.R.O., Hannah M.C., Jacobs J.L., Wales W.J., Moate P.J. (2019). Volatile fatty acids in ruminal fluid can be used to predict methane yield of dairy cows. Animals.

[35] Alcibahy M., Gafoor F.A., Mustafa F., El Fadel M., Al Hashemi H., Al Hammadi A., Al Shehhi M.R. (2025). Improved estimation of carbon dioxide and methane using machine learning with satellite observations over the Arabian Peninsula. Sci. Rep..

[36] Vivares G., Dijkstra J., Bannink A. (2025). Modeling diurnal rumen metabolism dynamics in dairy cattle: An update to a mechanistic model representing eating behavior, rumen content, rumination, and acid-base balance. J. Dairy Sci..

[37] Legaard C., Schranz T., Schweiger G., Drgoňa J., Falay B., Gomes C., Iosifidis A., Abkar M., Larsen P. (2023). Constructing neural network based models for simulating dynamical systems. ACM Comput. Surv..

[38] Jassim M.S., Coskuner G., Zontul M. (2022). Comparative performance analysis of support vector regression and artificial neural network for prediction of municipal solid waste generation. Waste Manag. Res..

[39] Odufuwa O., Tartibu L., Kusakana K. (2025). Artificial neural network modelling for predicting efficiency and emissions in mini-diesel engines: Key performance indicators and environmental impact analysis. Fuel.

[40] Louime C.J., Raza T.A. (2024). Development of artificial intelligence/machine learning (AI/ML) models for methane emissions forecasting in seaweed. Methane.

[41] Bukhari M.M., Alkhamees B.F., Hussain S., Gumaei A., Assiri A., Ullah S.S. (2021). An improved artificial neural network model for effective diabetes prediction. Complexity.

[42] Stamenković L.J., Antanasijević D., Ristić M., Perić-Grujić A., Pocajt V. (2015). Modeling of methane emissions using the artificial neural network approach. J. Serb. Chem. Soc..

[43] Liu J., Huang Q., Ulishney C., Dumitrescu C.E. (2022). Comparison of random forest and neural network in modeling the performance and emissions of a natural gas spark ignition engine. J. Energy Resour. Technol..

[44] Suraboyina S., Allu S.K., Anupoju G.R., Polumati A. (2022). A comparative predictive analysis of back-propagation artificial neural networks and non-linear regression models in forecasting seasonal ozone concentrations. J. Earth Syst. Sci..

[45] Wang Z., Peng X., Xia A., Shah A.A., Yan H., Huang Y., Zhu X., Zhu X., Liao Q. (2023). Comparison of machine learning methods for predicting the methane production from anaerobic digestion of lignocellulosic biomass. Energy.

[46] Wu Y.-C., Feng J.-W. (2018). Development and application of artificial neural network. Wirel. Pers. Commun..

[47] Negussie E., González-Recio O., Battagin M., Bayat A.R., Boland T., de Haas Y., Garcia-Rodriguez A., Garnsworthy P.C., Gengler N., Kreuzer M. (2022). Integrating heterogeneous across-country data for proxy-based random forest prediction of enteric methane in dairy cattle. J. Dairy Sci..

